# Stress and coping strategies among higher secondary and undergraduate students during COVID-19 pandemic in Nepal

**DOI:** 10.1371/journal.pgph.0001533

**Published:** 2023-02-15

**Authors:** Durga Rijal, Kiran Paudel, Tara Ballav Adhikari, Ashok Bhurtyal

**Affiliations:** 1 Institute of Medicine, Tribhuvan University, Maharajgunj, Kathmandu, Nepal; 2 Nepal Health Frontiers, Kathmandu, Nepal; 3 Department of Public Health, Section for Environment, Occupation and Health, Aarhus University, Aarhus, Denmark; 4 COBIN Project, Nepal Development Society, Bharatpur, Chitwan, Nepal; London School of Hygiene and Tropical Medicine Faculty of Epidemiology and Population Health, UNITED KINGDOM

## Abstract

Coronavirus Disease (COVID-19) pandemic has profoundly affected lives around the globe and has caused a psychological impact among students by increasing stress and anxiety. This study evaluated the stress level, sources of stress of students of Nepal and their coping strategies during the pandemic. A cross-sectional web-based study was conducted during the complete lockdown in July 2020 among 615 college students. Stress owing to COVID-19 and the lockdown was assessed using the Perceived Stress Scale (PSS), and Brief Coping Orientation to Problems Experienced (Brief COPE) was used to evaluate coping strategies. To compare the stress level among participants chi-square test was used. The Student’s *t-test* was used to compare Brief COPE scores among participants with different characteristics. The majority of study participants were female (53%). The mean PSS score was (±SD) of 20.2±5.5, with 77.2% experiencing moderate and 10.7% experiencing a high-stress level. Moderate to high levels of stress were more common among girls (92.6%) than boys (82.7%) (P = 0.001). However, there was a significant difference in perceived stress levels disaggregated by the students’ age, fields and levels of study, living status (with or away from family), parent’s occupation, and family income. The mean score for coping strategy was the highest for self-distraction (3.3±0.9), whereas it was the lowest for substance use (1.2±0.5). Students with a low level of stress had a higher preference for positive reframing and acceptance, whereas those with moderate to high levels of stress preferred venting. Overall, students experienced high stress during the lockdown imposed as part of governmental efforts to control COVID-19. Therefore, the findings of our study suggest stress management programs and life skills training. Also, further studies are necessary to conduct a longitudinal assessment to analyse the long-term impact of this situation on students’ psychological states.

## Introduction

Coronavirus disease (COVID-19) pandemic profoundly affected lives worldwide, which not only threatened physical health but global public health and social systems also collapsed during the coronavirus outbreak [1]. Evidence from the previous outbreaks of the severe acute respiratory syndrome (SARS) in 2003 and H1N1 influenza in 2009 illustrates that the community suffered considerable fear and panic, resulting in a significant psychological impact. A similar scenario was seen during the COVID-19 pandemic [2]. Higher levels of anxiety, worries, and social avoidance behaviors were confirmed in the general population in many studies conducted during the earlier pandemic of Middle East Respiratory Syndrome (MERS) [1, 3].

The effect of COVID-19 pandemic on global mental health is less studied. The increasing trend of this disease led to a global atmosphere of anxiety and depression due to disrupted travel plans, social isolation, media information overload, and panic buying of necessity goods and restrictions and economic shutdown imposed a complete change to the psychological environment of affected countries [4–6]. The infection has also caused a psychological impact among students by increasing stress and anxiety during the pandemic [7–9]. Studies showed a high prevalence of stress, anxiety, and depression among students during the COVID-19 pandemic as an effect of the disease itself and lockdowns. The reported stressors include delay in academic activities, financial difficulties, prolonged lockdown, overload of COVID-19 related information, home-schooling, fear of COVID-19 infection, and restrictive measures such as quarantine, isolation, and social distancing which caused an impact on psychological well-being [1, 6, 10, 11].

In Nepal, limited studies have been conducted to assess the psychological impact of COVID-19 among students. However, studies were conducted during the non-pandemic period, which revealed stress as a problem among the students, as 27% were stressed [12]. Similar to the studies conducted among students of various countries like Spain [7], China [11], and Turkey [13], students of Nepal also showed significant psychological impact during the pandemic [14]. According to the same study, 66.7% of students had some level of anxiety, with 27.1% having severe anxiety during the pandemic in Nepal [14].

To the best of our knowledge, there are no studies to assess the psychological effect and coping strategies among students other than medical students during the pandemic in Nepal. Students can make negative assessments of the pandemic and adopt various coping strategies that may affect their health and well-being. Additionally, there is a possibility that stress is a multi-faceted psychosocial experience that can affect different people differently; therefore, in addition to the prevalence of different levels of stress, it’s important to look at how it may be disproportionately impacting different groups of students. Therefore, a timely assessment of students’ mental health status and coping strategies may help reduce future negative consequences. Therefore, this study aimed to assess perceived stress levels, the sources of stress, and the coping strategies adopted by the students during the COVID-19 pandemic.

## Methods

### Study design and participants

A web-based descriptive, cross-sectional study was done in July 2020 during the complete lockdown in Nepal. The study was carried out among higher secondary and university-level undergraduate students. The study population was students in grades 11 and 12 of faculties, science, and management. Likewise, university-level students, only the bachelor-level students doing a four-year course on various subjects were included in the study. In our study, the undergraduate students were primarily in the fields of pure science, management, medical, paramedical, engineering/architecture, arts/humanities, and information technology. Our study was not confined to students of a specific college as we did not select a specific college for recruiting the participants.

### Sampling

To calculate the sample size, the expected proportion of stress of COVID-19 among the students was taken as 28.8% from a similar study conducted in China [9]. The sample size was calculated using the formula, n = z^2^pq/d^2^, where p = 0.29, q = 0.71, z = 1.96 at 95% confidence interval, and d = 0.05, which is 315. After assuming 10% of the non-response rate, the final calculated sample size was 347. A total of 643 responses were recorded, of which 28 were redundant, and only 615 were eligible for the analysis. Therefore, the sample size was 615.

### Study procedure

We followed the non-probability sampling technique, purposive and snowball sampling, and used our personal contact as well as several Facebook pages and groups of college students to invite them for the study. Google forms were disseminated among the students through email and social media platforms. Facebook groups, Facebook chat, and Viber were predominantly used to recruit participants. To limit the response from only the college students, forms were posted in the official Facebook groups of college students such as Nepal Public Health Student’s Society, institution-based Rotaract clubs, Facebook groups of students of other faculties, etc. and it was mentioned strictly that the forms should be filled only by the college students. Out of the total responses, only 16% of the responses came through email and personal invitation, and 84% of responses were from social media groups. Participation was voluntary; only those who ticked “I Agree” in the informed consent form, which was displayed on the front page of the questionnaire, could proceed further. Participants were allowed to fill out the form once, and multiple entries were not allowed.

### Ethical considerations

The study protocol was approved by the Institutional Review Committee of the Institute of Medicine, Tribhuvan University, Kathmandu, Nepal [Registration number: 85/ (6–11) E^2^/ 077/078]. Written digital consent was taken from study participants before completing the survey form. The informed consent form displayed on the front page of the form was for participants of age 18 and above. For the participants of age 16 and 17, it was mentioned in the consent form that the participants of this age group should fill up the form as per their parents’ consent.

### Measures

The online questionnaire contained three main parts. The first part included questions about socio-demographic characteristics, COVID-19, and sources of stress. Sources of stress were assessed by a question that included variables derived from the literature review. The second part was the Perceived stress scale. Perceived stress was assessed using the Perceived stress scale (PSS-10). A PSS is a 10-item questionnaire to measure the respondents’ self-reported stress level by assessing feelings and thoughts during the last month [15]. However, to focus on the scope of the study and to reflect on perceived stress during the pandemic, “experiences because of COVID-19” was added to each question. The Cronbach’s alpha value reported for this scale was 0.79 [16]. The PSS-10 consists of six positive items (items 1, 2, 3, 6, 9, and 10) and four negative items (4, 5, 7, and 8). In PSS, each question is rated on a 5-point Likert scale ranging from “Never (score 0)”, “Almost never (score 1)”, “Sometimes (score 2)”, “Fairly often (score 3)”, “Very often (score 4)” with a range of 0 to 40 for the total score of the scale. A higher level of stress is indicated by higher scores on this scale which is the score of 0–13 indicates “Mild stress”, 14–26 indicates “Moderate stress” and 27–40 indicates “High stress”. The scores for questions 4, 5, 7, and 8 were reversed, and the scores for perceived stress were calculated by summing the scores for the relevant items.

The third part consisted of the Brief COPE scale [17]. The original brief-COPE by Carver comprised 14 subscales, including self-distraction, active coping, denial, substance use, use of emotional support, use of instrumental support, behavioral disengagement, venting, positive reframing, planning, humor, acceptance, religion, and self-blame.” It is an abbreviated version of the COPE Inventory consisting of 28 items, two items in every 14 subscales, and each item is rated on a 4-point Likert Scale ranging from “I have not been doing this at all (score 1)”, “A little bit (score 2)”, “A medium amount (score 3)”, “I have been doing this a lot (score 4)”. The mean score of all items in each subscale is used, and a higher number indicates a higher preference for the coping strategies reported by the participants. Only ten dimensions of coping strategies were used.

### Data analysis

After completing data collection, responses stored in the web-based database (Google Drive) were downloaded, compiled, edited, and checked for errors in Microsoft Excel. Then the data was exported to Statistical Package for the Social Sciences (SPSS) version 20 for data cleaning, coding, and analysis. Continuous variables like coping strategies were expressed as mean and standard deviation (SD), whereas frequency and percentages were used to present categorical data. The chi-square test was used to assess the association of perceived stress levels among participants with different characteristics. The Student’s *t-test* for independent samples was used to compare the mean values of coping strategies in relation to studied variables.

## Results

### Socio-demographic characteristics

Table 1 depicts the socio-demographic characteristics of the respondents. Among the 615 respondents, the majority (53.0%) were female. The age ranged from 16 to 29, with a mean age (±SD) of 20.5 (±2.5). More than two-thirds (70.2%) of the respondents were Brahmin/Chhetri. The majority of the respondents (92.7%) were living with their family, and only 9.6% of the respondents disclosed having their family/friends/relatives infected with COVID-19 or in isolation (Table 1).

**Table 1:** Socio-demographic characteristics of respondents.

Variables (n=615)	Category	Number (n)	Percentage (%)
**Age (years)**	<=19	225	36.6
	>19	390	63.4
**Sex**	Male	289	47.0
	Female	326	53.0
**Ethnicity**	Brahmin/Chhetri	432	70.2
	Janajati	117	19.1
	Madhesi	49	8.0
	Others (Muslim, Dalit)	17	2.7
**Educational level**	Higher secondary	205	33.3
	Undergraduate	410	66.7
**Educational Background**	Pure Science	168	27.3
	Management	155	25.2
	Medical/Paramedical	208	33.8
	Engineering/Architecture	42	6.8
	Others	42	6.8
**Occupation of Parents**	Agriculture/Household	154	25.0
	Service	268	43.6
	Business/Foreign employment	193	31.4
**Average monthly income of the family (NRs)**	<20,000	184	29.9
	20,000-50,000	277	45.0
	>50,000	154	25.0
**Currently Status of living**	With family	570	92.7
	Without family	45	7.3
**Family/friends/relatives infected with COVID-19 or are in isolation**	No	556	90.4
	Yes	59	9.6

### Perceived stress

The majority of the students (77.2%) had a moderate level of perceived stress, whereas 12.0% had low perceived stress, and 10.7% had high perceived stress. The overall mean stress score (±SD) was 20.2 (±5.5). Among the socio-demographic variables, only gender was significantly associated with the level of stress. Male respondents were observed to have significantly less stress than female respondents (p-value = 0.001). Table 2 shows the association of the level of perceived stress with socio-demographic variables.

**Table 2:** Association of the level of perceived stress with socio-demographic variables.

Variables/Level of stress	Low Perceived Stress [n (%)]	Moderate to high perceived stress [n (%)]	p-value (χ^2^)
**Age Group**			
< = 19	25 (11.1)	200 (88.9)	0.594
>19	49 (12.6)	341 (87.4)	
**Gender**			
Male	50 (17.3)	239 (82.7)	0.001*
Female	24 (7.4)	302 (92.6)	
**Ethnicity**			
Brahmin/Chettri	57 (13.2)	375 (86.8)	0.394
Janajati	22 (18.8)	95 (81.2)	
Others (Madhesi included)	6 (9.0)	60 (91.0)	
**Educational Level**			
Higher secondary	21 (10.2)	184 (89.8)	0.335
Undergraduate	53 (12.9)	357 (87.1)	
**Educational Background**			
Pure Science	16 (9.5)	152 (90.5)	0.404
Management	17 (11.0)	138 (89.0)	
Medical/Paramedical	26 (12.5)	182 (87.5)	
Engineering/Architecture	7 (16.7)	35 (83.3)	
Others	8 (19.0)	34 (81.0)	
**Occupation of Parents**			
Agriculture/household	22 (14.3)	132 (85.7)	0.596
Service	31 (11.6)	237 (88.4)	
Business/foreign employment	21 (10.9)	172 (89.1)	
**Family income/month (NRs)**			
<20,000	30 (16.3)	154 (83.7)	0.073
20,000–50,000	31 (11.2)	246 (88.8)	
>50,000	13 (8.4)	141 (91.6)	
**Current Status of living**			
With Family	68 (11.9)	502 (88.1)	0.781
Without Family	6 (13.3)	39 (86.7)	
**Family/friends/relatives infected by COVID-19 or in isolation**			
No	70 (12.6)	486 (87.4)	0.274**
Yes	4 (6.8)	55 (93.2)	

*Significant (p<0.05)

**Continuity correction

### Sources of stress among students

The most important sources of stress reported by students were the long duration of lockdown (60.7%) and excessive hearing of news related to COVID-19 (50.1%). Out of 12 sources of stress, only one source, i.e., delay in the resumption of teaching/learning or fear of extension of the academic year, was significantly associated with the level of perceived stress.

### Coping strategies used by students

Out of the ten coping strategies, only three were significantly associated with perceived stress. Students with a low stress level had a higher preference for positive reframing and acceptance, whereas those with moderate to high levels of stress preferred venting more. (Table 3). Self-distraction was the highest used coping strategy, followed by acceptance, and substance use was the lowest (Table 4).

**Table 3:** Coping strategies and their association with the level of perceived stress.

Coping Strategy	Low perceived stress	Moderate to high perceived stress	p-value
	Mean± SD	Mean± SD	(t-test)
Positive reframing	2.7±1.0	2.4±0.9	0.029*
Venting	1.7±0.8	2.1±0.8	<0.001*
Self-distraction	3.4±0.8	3.3±0.9	0.2
Active coping	2.8±1.0	2.7±0.9	0.8
Religion	2.0±1.1	2.1±1.0	0.5
Humour	1.8±1.0	1.8±1.0	0.9
Use of emotional support	2.2±0.9	2.4±0.9	0.1
Substance use	1.1±0.5	1.2±0.5	0.9
Planning	2.9±0.9	2.8±0.9	0.1
Acceptance	3.3±0.8	3.0±0.8	0.007*

*Significant (p<0.05)

**Table 4:** Coping strategies and their association with gender.

Category	Overall	Gender		
	Mean± SD	Male	Female	p-value
		Mean± SD	Mean± SD	(t-test)
Self-distraction	3.3± 0.9	3.1± 1.0	3.4± 0.8	<0.001*
Acceptance	3.1± 0.8	3.0± 0.8	3.1± 0.8	0.04*
Planning	2.8± 0.9	2.7± 0.9	2.9± 0.9	0.03*
Active coping	2.7± 0.9	2.7± 0.9	2.7± 1.0	0.2
Use of emotional support	2.4± 0.9	2.2± 0.9	2.4± 0.9	<0.001*
Positive reframing	2.4± 0.9	2.5± 0.9	2.4± 0.8	0.1
Religion	2.1± 1	1.9± 0.9	2.3± 1.0	<0.001*
Venting	2.1± 0.8	1.9± 0.8	2.1± 0.8	0.003*
Humour	1.8± 1	1.8± 1.0	1.6± 0.9	<0.001*
Substance use	1.2± 0.5	1.2± 0.7	1.0± 0.2	<0.001*

*Significant (p<0.05)

Gender was observed to be one of the main factors where significant differences were observed for all the coping strategies used in the study except positive reframing (p = 0.1) and active coping (p = 0.2) (Table 4).

## Discussion

This study examined perceived stress among college and university students during the COVID-19 pandemic and the lockdown period in Nepal. Our study found that the majority of the students (77.2%) had moderate perceived stress, which resembles the findings of other studies from Spain [7], China [11], India [18], the US, and the UK [19] which reported a high level of mental health problems during the COVID-19 outbreak. Likewise, using the same measurement scale (i.e., PSS-10), in a study among students in Saudi Arabia during the COVID-19 outbreak, more than half of the participants (55%) showed moderate levels of stress, and 30.2% showed high stress [20]. Another study from Pune, India, reported that 82.6% of the students experienced moderate perceived stress, and a high perceived stress score was seen in 13.35% of the students during the COVID-19 pandemic [18]. Likewise, university students in southeast Serbia reported a mean perceived stress score of (20.3±7.6) which is similar to our findings (20.2±5.5) [21].

However, the result of our study contrasted with the findings from Turkey, in which 71.2% reported high perceived stress [13]. In the previous studies conducted in Nepal during the non-pandemic period, stress was found among 27% of students, 20.9% faced psychological morbidity, and the majority of the students (51%, n = 350) reported moderate to extremely severe levels of stress, anxiety, and depression [12, 22, 23]. In another study conducted during the non-pandemic period in Nepal, 60.4% of the students experienced moderate stress levels, and only 0.6% of the students experienced high-stress levels [24]. However, in our study, the percentage of students having stress was higher than in studies conducted during a non-pandemic situation in Nepal. Adding to it, the mean perceived stress score of (20.2±5.5) suggests that our participants had relatively high stress compared with established norms for a general population sample aged 18–29 (14.2±6.2) [15]. Furthermore, the perceived stress results in our study were relatively higher (20.2±5.5) than the results obtained in an earlier pre-COVID-19 survey among the Serbian students (14.9±6.3) [21]. Likewise, the perceived stress reported by the Malaysian study during the non-pandemic period was found to be relatively lower (46.3%) [25] than the result of our study, which may indicate that the pandemic might have aggravated the stress among students across the globe.

In this study, only gender was associated with the level of perceived stress. The female students were observed to have a higher mean score of perceived stress (21.0±5.1), similar to the findings from other studies conducted during the pandemic [13, 19, 20] that have shown significant gender differences in the psychological response to the pandemic. Likewise, the study results are in line with the recent studies carried out among the student of Spanish University [7] and the Saudi Arabian students [26], which showed significant gender differences. Therefore, high stress levels among females might have been attributed to various factors, including hormonal changes and expression of emotions and thoughts regarding their social situation [20], and the recent pandemic might have exacerbated this situation. Sociocultural inequity and gender norms, differences in the distribution of resources and restricted control over the economy make females more vulnerable to mental health problems in most of the low-and middle-income countries [27].

In Nepal, there is a difference in the socialization pattern of men and women [28]. Women are more likely to be more open about their feelings and admit their stress, whereas men are more reluctant to report psychological duress, which may lead to gender differences in terms of the appraisal process of stressful events [29]. In addition, women in Nepal have a higher societal expectation of being in caretaking roles, which may be even more stressful during a pandemic. Sometimes, they cannot fulfill the expectation of family members, so women are often abused and victimized [30]. In addition to economic hardship during the pandemic, a mental health risk for women could have been driven by gender-based violence (GBV) embedded in social norms [27]. The United Nations identified GBV as one of the areas of the impacts of COVID-19 on women [31]. During the COVID-19 lockdown in Nepal, several cases of domestic violence against women and girls were reported, which could directly affect their mental health [32]. Therefore, these gender differences open a path for more gender-specific intervention. The high prevalence of perceived stress and significant gender difference also suggests specific psychological measures prepared to prevent perceived stress and other mental health problems, especially for women.

In one of the studies, age and educational level were significantly associated with stress, where university students had a significantly higher mean score of perceived stress than intermediate and secondary school students. However, no such association was found in our study between perceived stress and age and perceived stress and educational level [20]. Similarly, in our research, there was no significant association between educational background and the stress level, contrary to other studies. Students in management-related studies seemed to have a higher level of anxiety than medical students during the pandemic [33]. This might be because students of all the faculties in our study could have been well-informed about the pandemic and the precautionary measures.

Likewise, the parents’ occupation did not affect the level of perceived stress, unlike the findings of the previous study [34]. In one of the studies conducted among college students in China, anxiety regarding the epidemic was associated with the source of parent income, whether living with parents and whether a relative or an acquaintance was infected with COVID-19, which is not in agreement with the results of our study [11].

Given the above findings, students considered many factors as sources of stress during the pandemic. More than six out of ten study participants considered the long duration of the lockdown as the major source of stress. It corroborates with the literature suggesting that lockdown is one of the important stressors during COVID-19 and has a considerable psychological impact on the well-being of people [6]. This might be because, during the lockdown, outdoor activities were hampered. Likewise, half of the students (50.1%) indicated stress induced by news outlets, similar to those found among the US College students. This type of stress may be exacerbated by a large amount of misinformation, including false and fabricated information distributed through news and social media [35]. Also, studies suggest that people may develop “headline stress disorder” during the modern pandemic, which is characterized by stress to endless reports from the news media [36].

Similarly, in our study, students considered a delay in the resumption of teaching/learning or fear of extension of the academic year, fear of contracting the virus by family/friends/relatives, financial difficulties, worries of the future like employment, gaining weight, interpersonal conflict with roommate/family members as other important sources of stress which resembles with the recent findings [35, 37]. However, our findings related to an overload of the assignment were different from that found in the recent study in which 66.6% considered increased class workload as the source of stress. In contrast, in our study, only 11.1% considered it the source of stress [35]. This might be because colleges and universities were closed; only limited colleges and universities ran virtual classes. In the study conducted among university students in Pakistan, major distress was related to restricted social meetings with friends (84.7%) and fear of family/friends getting infected (70.9%), but in our study, only 14.1% and 44.6% of university-level undergraduate students considered inability to meet family/friends and fear of family/friends/relatives being infected as the source of stress respectively [38]. This might be because most of the students were with their family/relatives during the period of lockdown, and the virus may not be present at the community level in their place of residence.

To cope with the stressors, students used various coping strategies in our study. The mean scores for active coping strategies (acceptance, planning, active coping, positive reframing, use of emotional support) were greater than avoidant coping strategies (venting, substance use), as well as religious coping and humor except for self-distraction. A similar result was found in the study conducted among Pakistani students, where all the active coping strategies had higher mean scores [38]. Our study found that substance use was the least common coping strategy among the students, as in the previous studies. However, the average score of substance use was found to be 1.2± 0.5 in our study, and the average score of substance use was reported as 2.5±1.0 and 2.7±1.4 from the same study conducted in Nepal and Malaysia, respectively, during the non-pandemic period which suggests that the average score of substance use was less during the pandemic period [22, 25]. This indicates that substance use as a coping strategy for stress might have decreased during the pandemic as the shops selling these products were closed following the government rule of lockdown.

Similarly, living with parents/family members during the lockdown and getting adequate emotional support might also be a reason for decreased substance use by the students. In our study, students with moderate to high-stress levels preferred venting more than the students who perceived low stress. However, students with a low stress level had a higher preference for positive reframing and acceptance. Mixed use of both the active and the avoidant types of coping strategies might be because, in times of uncontrollable situations and diverse types of stressors, any type of coping might be helpful in reducing stress. In our study, active coping was not associated with stress level; this might be because of the uncertainty and uncontrollability of COVID–related stressors.

Likewise, religious coping was also not associated with the level of stress, which is consistent with the finding of a previous study conducted during the non-pandemic period [25]. Nevertheless, this result contradicts the recent finding that shows religious coping as the most effective coping strategy to deal with severe stress and practiced by many severely stressed students during the pandemic [26]. This might be because, in our study, the study population was a younger group of people who tend to adopt other coping measures rather than religious coping. The older students in our study used positive reframing more than the teens, which resembles the previous study’s findings [25].

Our study found the association of gender with self-distraction, planning, humor, acceptance, and religious coping. Male students used self-distraction, acceptance, and religious coping less than females, and females used humor coping less than males, according to a recent study. However, the result of our study differed in planning; males used planning more than females in our study, which contrasts with the previous study [38]. In our study, male students used active coping less and substance use more than female students, resembling the previous study’s findings [25]. In the previous study conducted during the outbreak, the mean score was higher for religious coping among university students. However, the mean score was the highest for self-distraction among undergraduate students in our study, followed by acceptance. In contrast, it was the lowest for substance use, similar to the previous findings [38]. Furthermore, there might be many reasons behind such findings. One of the reasons might be having many options like watching TV, reading books, using social media, playing online games, attending online classes, etc., as there was a lockdown and low substance use might be because students were living with their family/relatives and there was no access of substance due to lockdown. In another study conducted among undergraduate medical students, commonly used coping strategies were “regular exercise”, “watching online movies and playing online games”, “religious activities,” and “learning to live in a COVID-19 situation and accept it” which resembles active coping, self-distraction, religious coping, and acceptance and these strategies were also, commonly used by the medical students in our study [26].

Therefore, the findings of our study suggest stress management programs as well as life skills training and mindfulness therapy, which have been validated to reduce stress and anxiety [39, 40]. Similarly, regular exercise and good sleep are recommended, which have been found to have mitigating effects on negative emotions without social, medical burden [9, 41]. Even though female students presented higher stress levels, providing mental health support systems and promoting physical activity regularly is necessary for all students, which could decrease perceived stress levels. Online training, workshops, and contests for the students from the respective educational institutions can also be conducted to distract them from stressful situations, reduce stress, and protect them from future psychological consequences. Therefore, further studies are necessary to conduct a longitudinal assessment to analyse the long-term impact of this situation on students’ psychological states and to enable more robust evidence on causal links and pathways.

## Strengths and limitations

There are certain limitations in this study. First, the study was conducted during the peak time when COVID-19 was spreading rapidly, so the study used self-reported questionnaires, which may have issues with subjectivity and reliability. However, respondents were assured of the anonymity of the data. Similarly, social desirability bias and lack of conscientious response in respondents may limit the accuracy of the present findings. Furthermore, the findings from the self-reported measures of mental health cannot be subjected to direct treatment without using diagnostic tools. However, the self-reported tools are easy and useful for assessing individual perceptions of their illness. The PSS cut-offs are the ones established in the literature and may not be the best way to capture the variation in stress expressed in this sample. Second, the study might not represent the population with no access to the internet. Third, the questionnaire was in English, which might have created a language barrier. Fourth, the study used only ten Brief COPE dimensions and missed other dimensions that the respondents could have manifested. Fifth, the limited sample size and purposive sampling approach findings may not represent the entire student population. Sixth, the study was cross-sectional under an unprecedented situation and had a limitation in determining a causal relationship between factors of interest and perceived stress and evaluating the stress level during the actual pandemic and pre-pandemic period. In addition, there may be an exacerbation of existing psychiatric illness, substance use, etc., so the findings here do not represent the only impact of the disease on mental health. Despite the limitations of this study related to web-based cross-sectional design with self-reported measures, the findings add new evidence regarding stress among students during the COVID-19 pandemic. It could also be a piece of baseline evidence for future work on stress and coping strategies among students in Nepal.

## Conclusion

The study has shown that the COVID-19 pandemic caused a significant impact on the mental health of the students of Nepal as the majority (87.9%) of the students had moderate to a high level of perceived stress, where only gender was significantly associated with a level of stress. Delay in a resumption of teaching/learning or fear of extension of the academic year was the major source of stress associated with the level of perceived stress. Furthermore, the major coping strategies adopted by the students were self-distraction, acceptance, and active coping. Therefore, these results could be used as a baseline to find the extent of the impact of COVID-19 on the mental health of the students in Nepal. Thus, public attention should be given to the high prevalence of perceived stress and the significant gender differences, and a certain psychological intervention should focus on women to prevent perceived stress and other mental problems in women.

## Supporting information

S1 Table Level of perceived stress among students. (DOCX)

S2 Table Sources of stress among students. (DOCX)

S3 Table Association of the level of stress with sources of stress. (DOCX)

S1 Data Data underlying the result of the study. (XLS)
