# Supplementary material for: Stress and coping strategies among higher secondary and undergraduate students during COVID-19 pandemic in Nepal
Source: PLOS Glob Public Health. 2023 Feb 15;3(2):e0001533. doi: 10.1371/journal.pgph.0001533 (PMC10021748; doi:10.1371/journal.pgph.0001533)
Supplement: S1 Table — (DOCX) [file pgph.0001533.s001.docx]

**S1 Table: Level of perceived stress among students (n=615)**

| **Level of Stress** | **Number (n)** | **Percentage (%)** | **Higher secondary**  **[n=205 (%)]** | **Undergraduate**  **[n=410**  **(%)]** |
| --- | --- | --- | --- | --- |
| Low stress (0-13)  Moderate stress (14-26)  High stress (27-40)  Mean stress score (20.2±5.5) | 74  475  66 | 12.0  77.2  10.7 | 21 (10.2)  161 (78.5)  23 (11.2) | 53 (13.0)  314 (76.5)  43 (10.5) |
