# Supplementary material for: Stress and coping strategies among higher secondary and undergraduate students during COVID-19 pandemic in Nepal
Source: PLOS Glob Public Health. 2023 Feb 15;3(2):e0001533. doi: 10.1371/journal.pgph.0001533 (PMC10021748; doi:10.1371/journal.pgph.0001533)
Supplement: S2 Table — (DOCX) [file pgph.0001533.s002.docx]

**S2 Table: Sources of stress among students (n=615)**

| **Source of stress** | **Overall**  **[n, (%)]** | **Higher secondary students (n=205)**  **(%)** | **Undergraduate (n=410)**  **(%)** |
| --- | --- | --- | --- |
| Long duration of lockdown | 373 (60.7) | 108 (52.7) | 265 (64.6) |
| Excessive hearing of news related to COVID-19 | 308 (50.1) | 97 (47.3) | 211 (51.5) |
| Delay in resumption of teaching/learning Or fear of extension of academic year | 307 (49.9) | 84 (41.0) | 223 (54.4) |
| Fear to contract virus by oneself/family/friends/relatives | 269 (43.7) | 86 (42.0) | 183 (44.6) |
| Uncertainty of board exams | 264 (42.9) | 112 (54.6) | 152 (37.1) |
| Worries of the future like employment | 240 (39.0) | 59 (28.8) | 181 (44.1) |
| Financial difficulties | 239 (38.9) | 82 (40.0) | 157 (38.3) |
| Gaining weight during lockdown | 116 (18.8) | 39 (19.0) | 77 (18.8) |
| Lack of internet to attend online classes | 114 (18.5) | 38 (18.5) | 76 (18.5) |
| Interpersonal conflict with roommate/family member | 86 (14.0) | 26 (12.7) | 60 (14.6) |
| Living away from home and/or inability to meet family/friends/relatives | 72 (11.7) | 14 (6.8) | 58 (14.1) |
| Overload of assignments | 68 (11.1) | 19 (9.3) | 49 (12.0) |
