# Supplementary material for: Stress and coping strategies among higher secondary and undergraduate students during COVID-19 pandemic in Nepal
Source: PLOS Glob Public Health. 2023 Feb 15;3(2):e0001533. doi: 10.1371/journal.pgph.0001533 (PMC10021748; doi:10.1371/journal.pgph.0001533)
Supplement: S3 Table — (DOCX) [file pgph.0001533.s003.docx]

**S3 Table: Association of the level of stress with sources of stress**

| **Source of stress** | **Overall**  **(n, %)** | **OR** | **95% CI** | **p-value**  **(χ^2^)** |
| --- | --- | --- | --- | --- |
| Long duration of lockdown | 373 (60.7) | 1.4 | 0.9-2.3 | 0.1 |
| Excessive hearing of news related to COVID-19 | 308 (50.1) | 1.4 | 0.8-2.2 | 0.2 |
| Delay in resumption of teaching/learning Or fear of extension of the academic year | 307 (49.9) | 1.7 | 1.1-2.9 | 0.02^*^ |
| Fear to contract virus by the family members/oneself/friends/relatives | 269 (43.7) | 1.5 | 0.9-2.5 | 0.1 |
| Uncertainty of board exams | 264 (42.9) | 1.2 | 0.7-1.9 | 0.4 |
| Worries of the future | 240 (39.0) | 1.6 | 0.9-2.7 | 0.08 |
| Financial difficulties | 239 (38.9) | 0.9 | 0.6-1.5 | 0.7 |
| Gaining weight during lockdown | 116 (18.8) | 1.4 | 0.7-2.7 | 0.3 |
| Lack of internet to attend online classes | 114 (18.5) | 0.9 | 0.5-1.8 | 0.9 |
| Interpersonal conflict with roommate/family member | 86 (14.0) | 1.9 | 0.8-4.6 | 0.1 |
| Living away from home and/or inability to meet family/friends/relatives | 72 (11.7) | 0.6 | 0.3-1.1 | 0.09 |
| Overload of assignments | 68 (11.1) | 0.6 | 0.3-1.1 | 0.1 |
